# Supplementary material for: Observation of a single protein by ultrafast X-ray diffraction
Source: Light Sci Appl. 2024 Jan 12;13:15. doi: 10.1038/s41377-023-01352-7 (PMC10786860; doi:10.1038/s41377-023-01352-7)
Supplement: Supplementary file 1 — Supplementary Information [file 41377_2023_1352_MOESM1_ESM.pdf]

Observation of a single protein by ultrafast X-ray diffraction

Tomas Ekeberg[a], Dameli Assalauova[b], Johan Bielecki[c], Rebecca Boll[c], Benedikt J. Daurer[d], Lutz A. Eichacker[e], Linda E. Franken[f], Davide E. Galli[g], Luca Gelisio[b], Lars Gumprecht[h], Laura H. Gunn[a,i], Janos Hajdu[a], Robert Hartmann[j], Dirk Hasse[a], Alexandr Ignatenko[b], Jayanath Koliyadu[c,k], Olena Kulyk[l], Ruslan Kurta[c], Markus Kuster[c], Wolfgang Lugmayr[m,n], Jannik Lübke[h,o,p], Adrian P. Mancuso[c,q], Tommaso Mazza[c], Carl Nettelblad[r], Yevheniy Ovcharenko[c], Daniel E. Rivas[c], Max Rose[b], Amit K. Samanta[h], Philipp Schmidt[c], Egor Sobolev[c,s], Nicusor Timneanu[t], Sergej Usenko[c], Daniel Westphal[a], Tamme Wollweber[o,p,u,v], Lena Worbs[h,p], P. Lourdu Xavier[c,h,u], Hazem Yousef[c], Kartik Ayyer[o,u,v], Henry N. Chapman[h,o,p], Jonas A. Sellberg[k], Carolin Seuring[m,w], Ivan A. Vartanyants[b], Jochen Küpper[h,o,p], Michael Meyer[c], Filipe R.N.C. Maia[a,x] *

[a] Laboratory of Molecular Biophysics, Department of Cell and Molecular Biology, Uppsala University, Husargatan 3 (Box 596), SE-75124, Uppsala, Sweden

[b] Deutsches Electronen-Synchrotron DESY, Notkestrasse 85, D-22607 Hamburg, Germany

[c] European XFEL, Holzkoppel 4, 22869 Schenefeld, Germany

[d] Diamond Light Source, Harwell Science & Innovation Campus, Didcot OX11 0DE, UK

[e] University of Stavanger, Centre Organelle Research, Richard-Johnsensgate 4, 4021 Stavanger, Norway

[f] Leibniz Institute for Experimental Virology (HPI), Centre for Structural Systems Biology, Notkestraße 85, 22607 Hamburg, Germany

[g] Dipartimento di Fisica "Aldo Pontremoli", Università degli Studi di Milano, via Celoria 16, 20133 Milano, Italy

[h] Center for Free-Electron Laser Science, DESY, 22607 Hamburg, Germany

[i] Plant Biology Section, School of Integrative Plant Science, Cornell University, Ithaca, NY 14853, USA

[j] PNSensor GmbH, Otto-Hahn-Ring 6, D-81739 München, Germany

[k] Biomedical and X-Ray Physics, Department of Applied Physics, AlbaNova University Center, KTH Royal Institute of Technology, SE-10691 Stockholm, Sweden

[l] ELI Beamlines/ IoP Institute of Physics AS CR, v.v.i., Na Slovance 2, 182 21 Prague 8, Czech Republic

[m] Multi-User CryoEM Facility, Centre for Structural Systems Biology, Notkestr.85, 22607 Hamburg, Germany

[n] University Medical Center Hamburg-Eppendorf (UKE), Martinistrasse 52, 20246 Hamburg, Germany

[o] The Hamburg Center for Ultrafast Imaging, Universität Hamburg, Luruper Chaussee 149, 22761 Hamburg, Germany

[p] Department of Physics, Universität Hamburg, Luruper Chaussee 149, 22761 Hamburg, Germany

[q] Department of Chemistry and Physics, La Trobe Institute for Molecular Science, La Trobe University, Melbourne, Victoria 3086, Australia,

[r] Division of Scientific Computing, Science for Life Laboratory, Department of Information Technology, Uppsala University, Box 337, SE-75105 Uppsala, Sweden

[s] European Molecular Biology Laboratory, c/o DESY, Notkestrasse 85, 22607 Hamburg, Germany

[t] Department of Physics and Astronomy, Uppsala University, Box 516, SE-75120, Uppsala, Sweden

[u] Max Planck Institute for the Structure and Dynamics of Matter, Luruper Chaussee 149, 22761 Hamburg, Germany

[v] Center for Free-Electron Laser Science, Luruper Chaussee 149, 22761 Hamburg, Germany

[w] Department of Chemistry, Universität Hamburg, 20146 Hamburg, Germany

[x] NERSC, Lawrence Berkeley National Laboratory, Berkeley, CA 94720, USA

*E-mail: filipe.maia@icm.uu.se

Supplementary Information

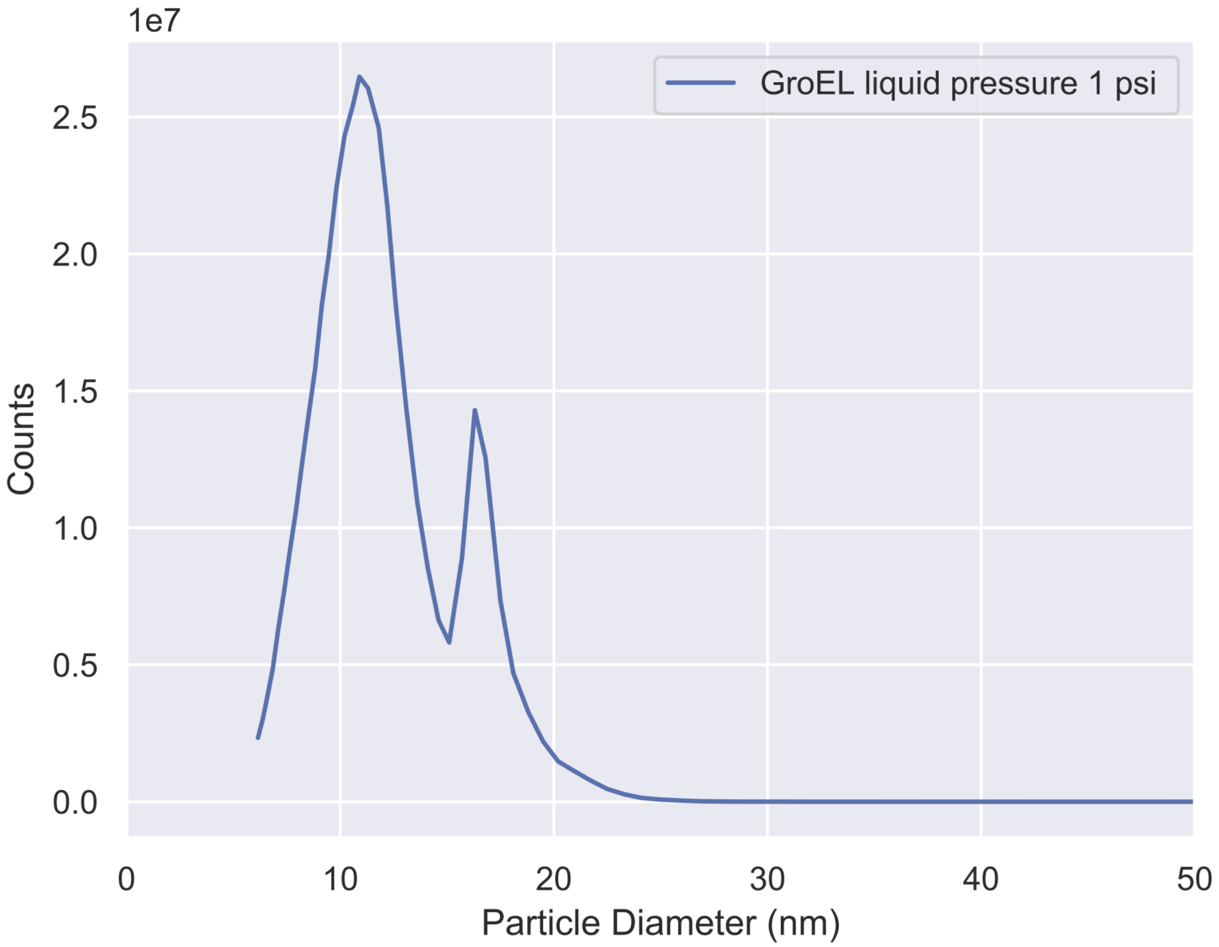

Figure S1 | Differential mobility analyzer(DMA) measurement of the sample. Spectrum of the particle diameters measured by the DMA, when running the electrospray in the same conditions that gave rise to the patterns described. The peak around 11 nm corresponds to the impurities in droplets without GroEL while the peak around 16 nm corresponds to a droplet with a GroEL complex.

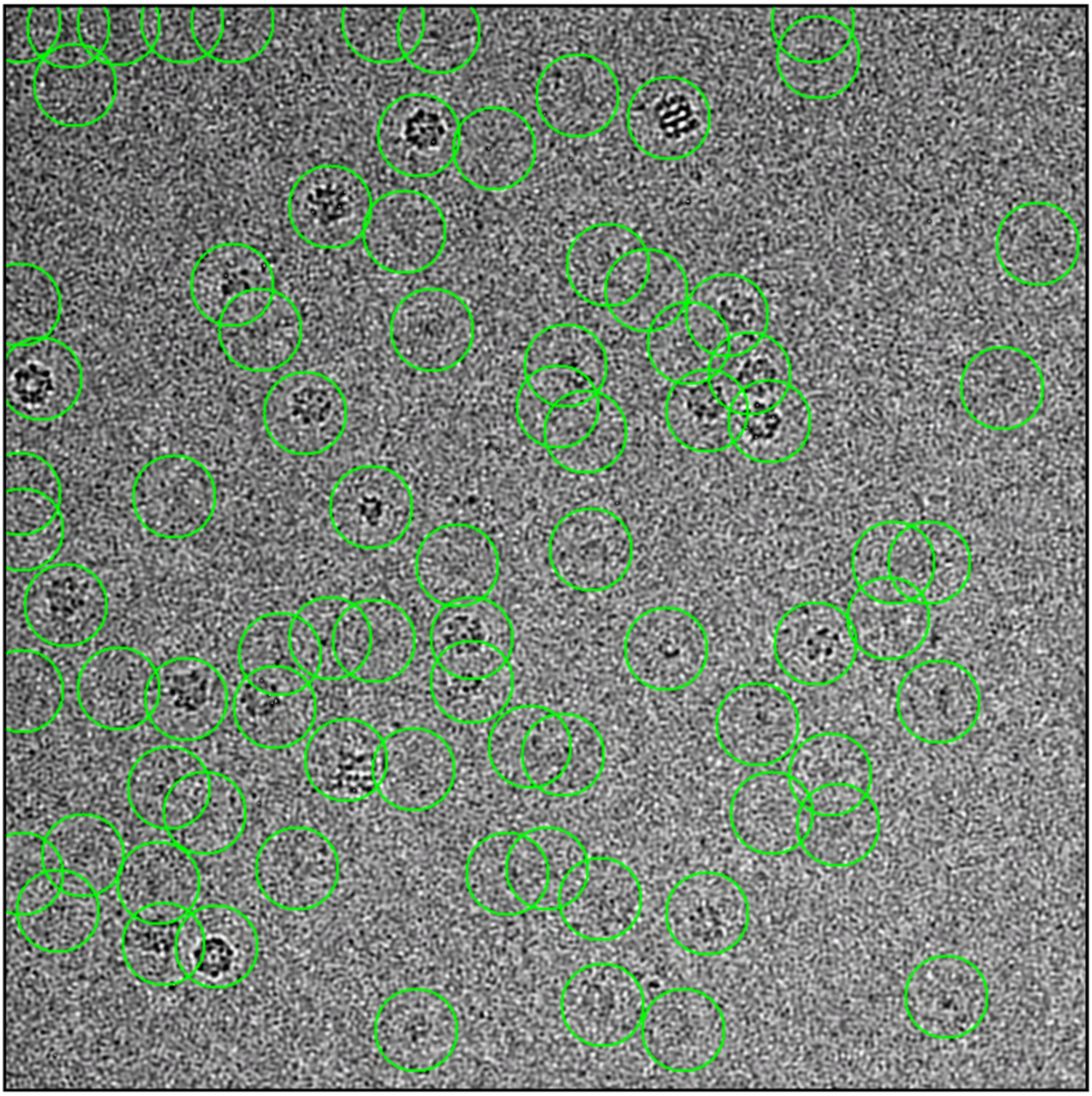

Figure S2 | Particle Selection based on the Laplacian Picking algorithm is shown in an example micrograph. Reference free particle selection with the Laplacian Picking algorithm from Relion 3.1 picks all intensities between 12 and 90 nm. The green circle is 300 Å in diameter. As can be seen in this example micrograph, most particles are picked as well as several small intensities in the background which can be noise or small proteins. This demonstrates unbiased picking, ideal for sample quality analysis.

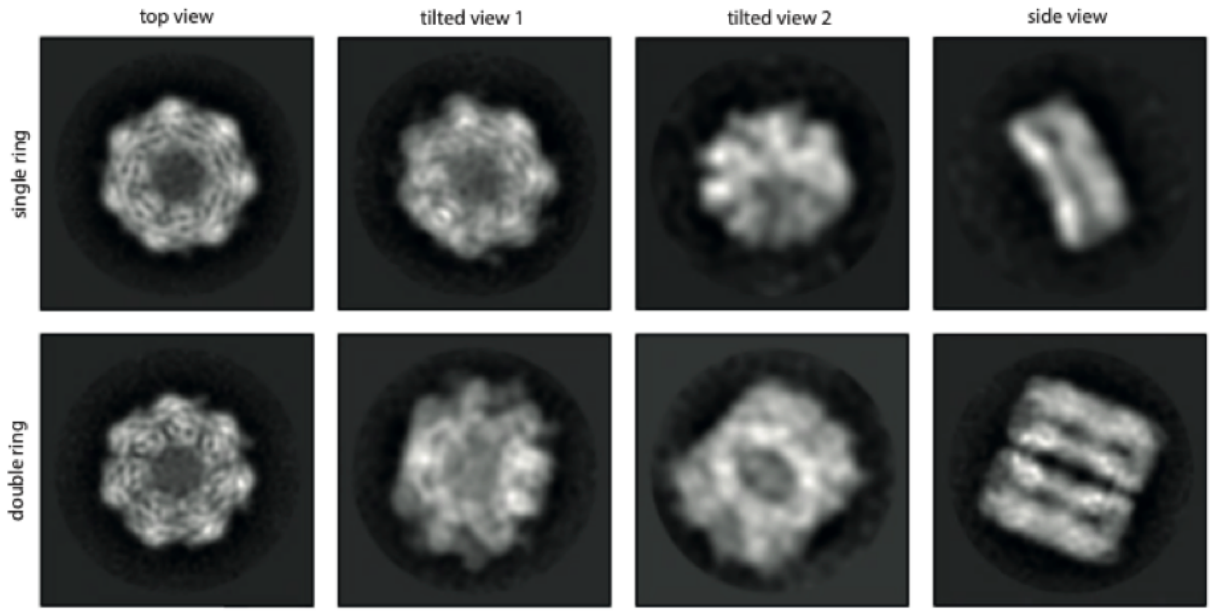

Figure S3 | Representative 2D class averages from Cryo-EM images of the sample. Sample quality analysis shows the presence of single- and dual-ring GroEL complexes. Eight representative 2D class averages from Cryo-EM images of the sample are shown. From projection images, it cannot be concluded whether these two different top views correspond to single-ring or dual-ring complexes. 2D cleaning of the picked dataset (**Fig. S2**) removed most of the particles that contained small protein or noise, and subsequent 3D heterogeneous refinement against a single- and a dual-ring reference, followed by further 2D cleaning allowed us to reduce the dataset to 3454 particles, of which 869 particles (32%) and 2676 particles (68%) were assigned to single- or dual-ring complexes respectively.

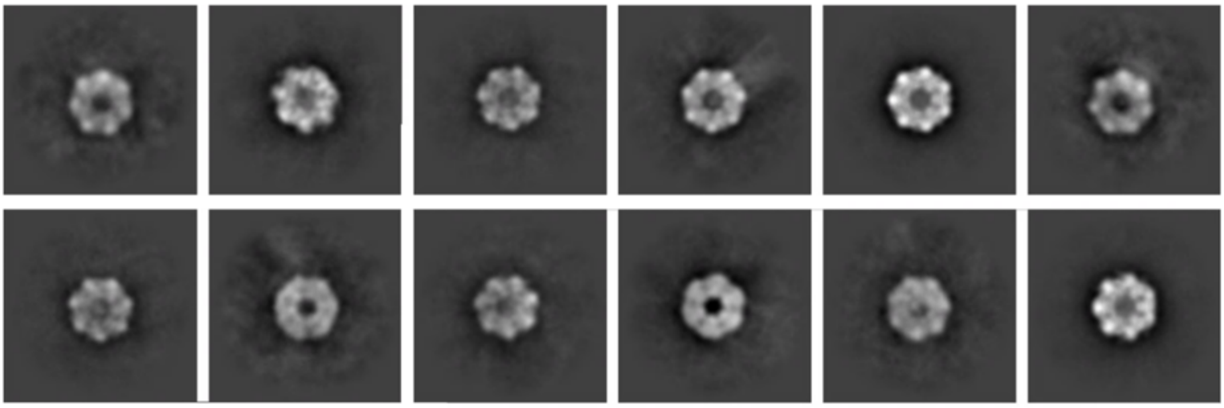

Figure S4 | Gallery of top view 2D classes corresponding to dual-ring GroEL. Gallery of top view 2D classes corresponding to dual-ring GroEL. Images were selected from the last round of 2D cleaning after heterogeneous refinement from the subset of particles corresponding to the GroEL dual-ring. Density can be observed in the centre of several classes, likely from attached proteins.

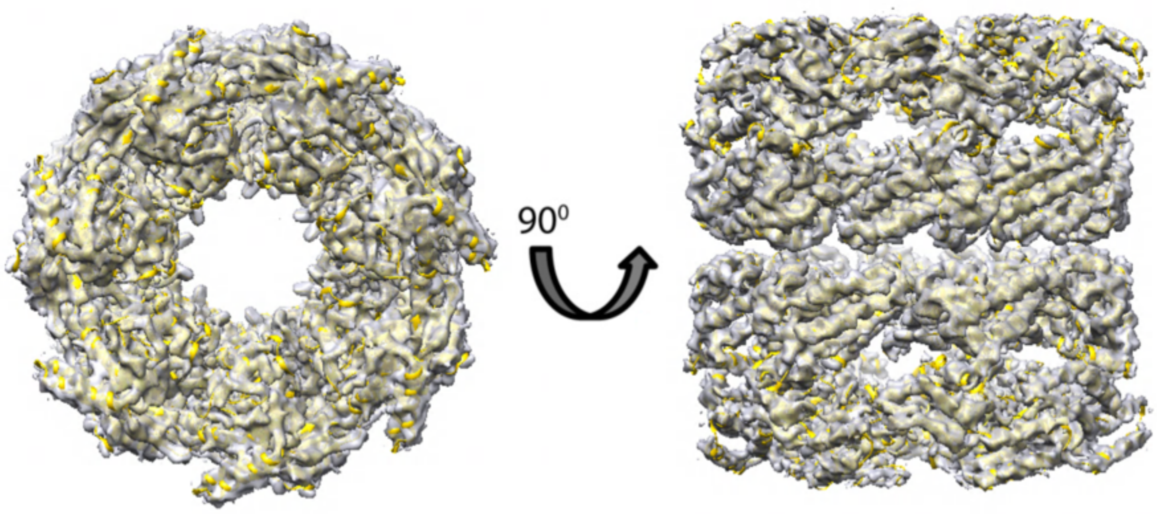

Figure S5 | Final 3D map at 4.6 Å resolution fitted to the PDB structure (5W0S). Final 3D map of GroEL dual-ring protein at 4.6 Å resolution with PDB structure (5W0S) fitted inside. No apparent differences were observed between map and model. As D7 symmetry was enforced during the 3D reconstruction, details that do not comply with the symmetry group, including any density within the rings, were averaged out and became invisible in the final 3D density.

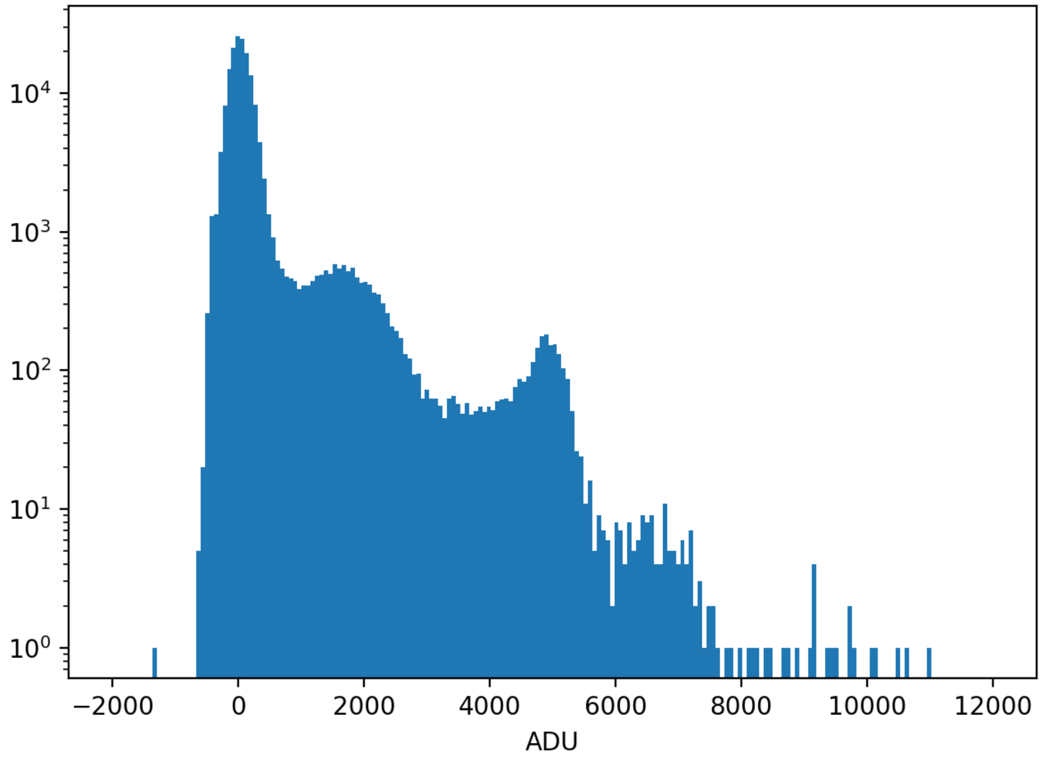

Figure S6 | Histogram of the detector signal for a single pixel. The sharp peak around 5000 ADU corresponds to a single X-ray photon at 1200 eV. The broad peak around 2000 ADU corresponds to the fluorescence signals from the $K_{\alpha 1}$-shells of carbon (277 eV), nitrogen (392 eV) and oxygen (525 eV).

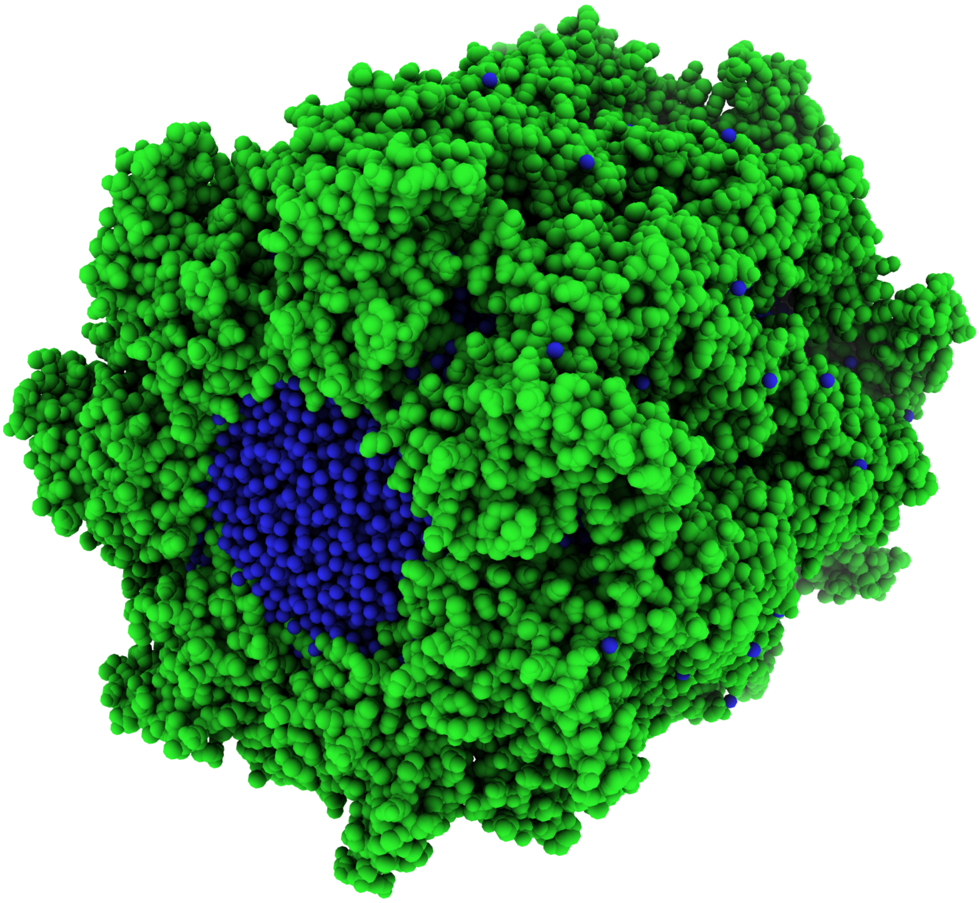

Figure S7 | Orientation of the GroEL in the X-ray beam. Model of GroEL in the orientation that best matches the experimental diffraction, corresponding to the orientation of the density model 3 that gives rise to the diffraction pattern in **Fig. 4b**. The X-ray beam direction is defined to be directly into the page.

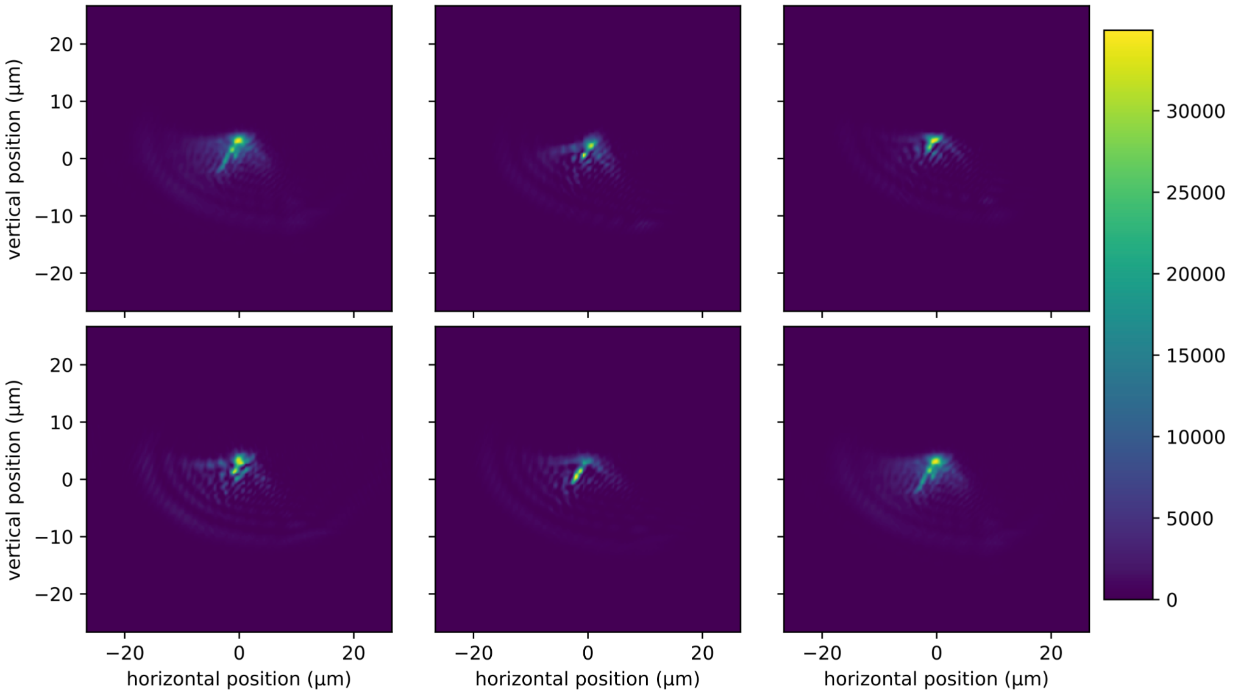

Figure S8 | Focal plane intensity distribution of different shots. Single-shot intensity distribution, of the focal plane, of five different shots. The bottom right corresponds to the average of the five shots. There was a variation in the focal plane intensity distribution from shot to shot, with an associated change in the focal spot. From the average intensity, the full width at half maximum of the focus was estimated at 1.7 μm x 2.3 μm. The focal plane intensity distribution was determined using the beamline Hartmann-type wavefront sensor (1).

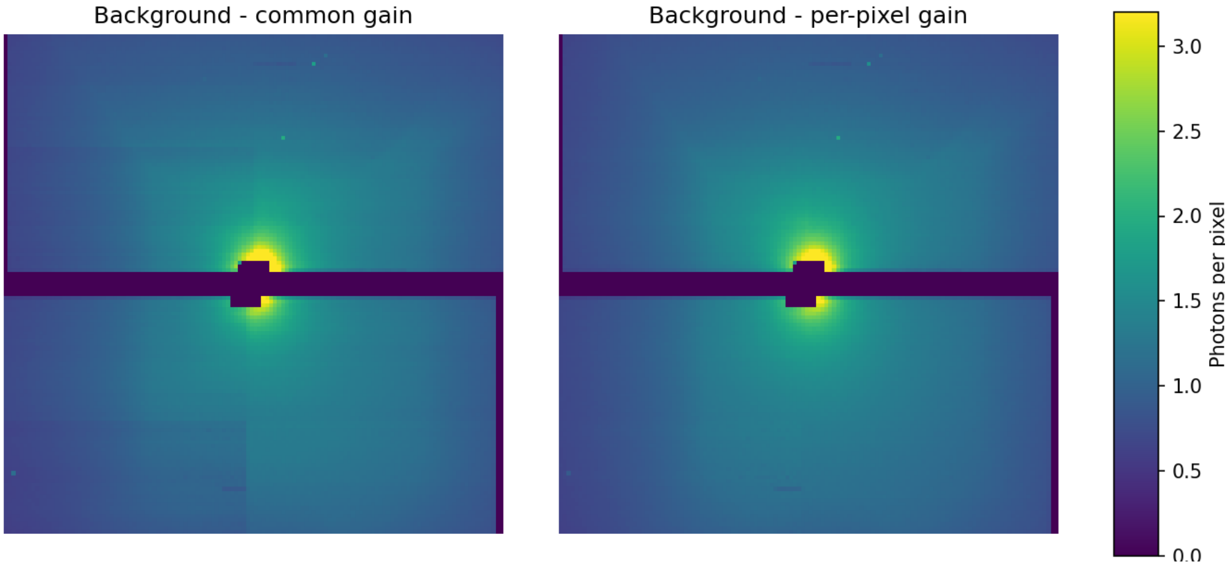

Figure S9 | Average photon background with and without per-pixel gain. When assuming the same gain for all the pixels, the boundaries between different ASICs in the detector are visible, as shown on the left-hand side. By calibrating the gain of each pixel, using pixel-level histograms, one obtains a smooth image with no visible boundaries, as shown in the right, which matches the expectation of what the gas background should look like.

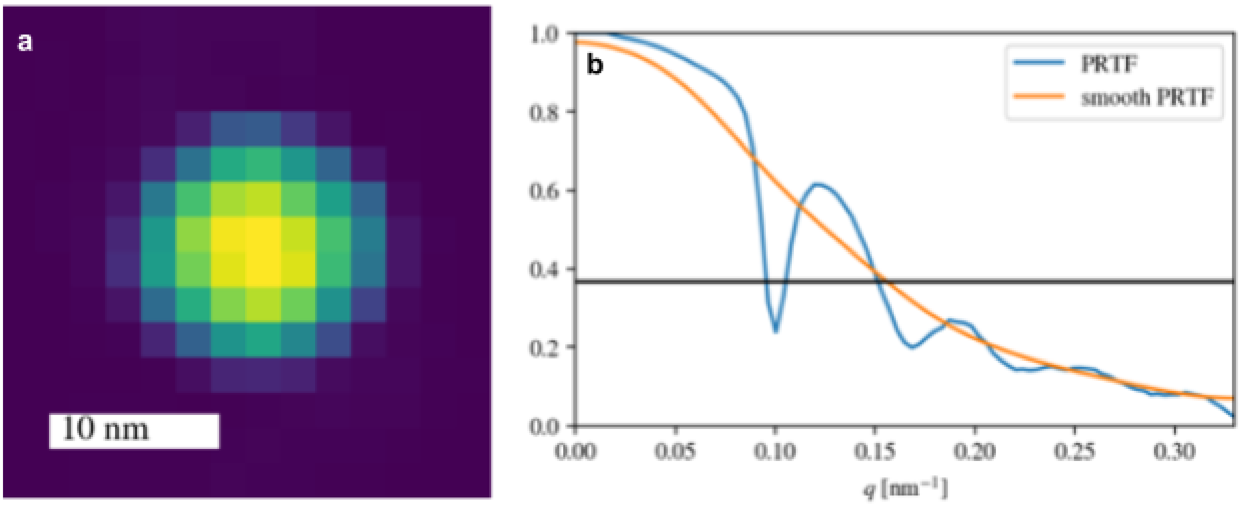

Figure S10 | Phase retrieval. a, The phase retrieved electron-density map. The phase-retrieval was performed using the relaxed averaged alternating projectors (RAAR) algorithm using positivity and reality constraints. The support was a circle with a diameter of 27 nm and was gradually decreased using the shrinkwrap method. The reconstruction was repeated 100 times and the presented map is the average reconstruction. **b,** The phase retrieval transfer function (PRTF) of the sample shows a successful reconstruction up to 0.16 nm^{-1}, corresponding to a resolution of 6.4 nm. The reconstruction matches the size of GroEL

but beyond that, the resolution is too low to allow any conclusions to be drawn about the structure.

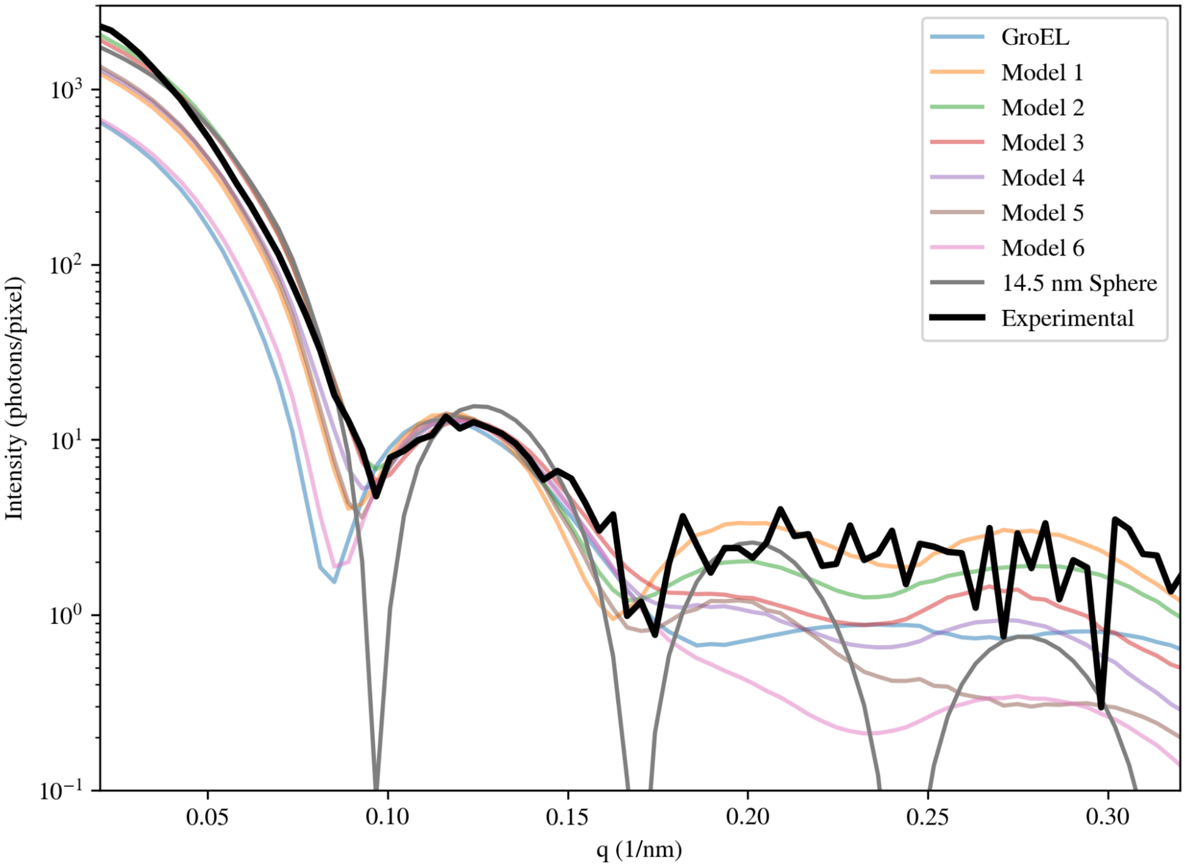

Figure S11 | Powder pattern comparison. The black line shows the sum of the 133 patterns within the size range 10 nm to 20 nm, excluding the single pattern used for detailed analysis. This data is compared to the simulated powder patterns of respectively a dry GroEL, the hydrated models, and the best fitting sphere (14.5 nm). All the lowly hydrated models provide a better fit than both the dry GroEL and the sphere, with model 2 providing the best fit. This suggests that the dataset includes many diffraction patterns from GroEL even though most of them are too weak for a more detailed analysis.

Bibliography

1. Mazza T, Baumann TM, Boll R, De Fanis A, Grychtol P, Ilchen M, et al. The beam transport system for the Small Quantum Systems instrument at the European XFEL: optical layout and first commissioning results. J Synchrotron Radiat. 2023 Mar 1;30(Pt 2):457–67.
